# A Model for Assessing Necessary Conditions for Rural Health Care’s Mobile Health Readiness: Qualitative Assessment of Clinician-Perceived Barriers

**DOI:** 10.2196/11915

**Published:** 2019-11-08

**Authors:** Bryan Weichelt, Casper Bendixsen, Timothy Patrick

**Affiliations:** 1 Marshfield Clinic Research Institute National Farm Medicine Center Marshfield, WI United States; 2 University of Wisconsin-Milwaukee Health Informatics Milwaukee, WI United States

**Keywords:** mHealth, clinician, physician, rural, patient, mobile health, health care

## Abstract

**Background:**

Mobile health (mHealth) technology dissemination has penetrated rural and urban areas alike. Yet, health care organization oversight and clinician adoption have not kept pace with patient use. mHealth could have a unique impact on health and quality of life for rural populations. If organizations are prepared to manage mHealth, clinicians may improve the quality of care for their patients, both rural and urban. However, many organizations are not yet prepared to prescribe or prohibit third-party mHealth technologies.

**Objective:**

This study explored organizational readiness for rural mHealth adoption, the use of patient-reported data by clinical care teams, and potential impact on improving rural health care delivery.

**Methods:**

Semistructured, open-ended interviews were used to investigate clinicians’ current practices, motivators, and perceived barriers to their use of mHealth technologies in rural settings.

**Results:**

A total of 13 clinicians were interviewed, and 53.8% (7/13) reported encouraging use of mHealth apps or wearable devices with rural patients. Perceived barriers to adoption were categorized into three primary themes: (1) personal (clinician), (2) patient, and (3) organizational. Organizational was most prominent, with subcodes of time, uniformity, and policy or direction. Thematic analysis revealed code-category linkages that identify the complex nature of a rural health care organization’s current climate from a clinician’s perspective. A thematic map was developed to visualize the flow from category to code. Identified linkages guided the development of a refined rural mHealth readiness model.

**Conclusions:**

Clinicians (including physicians) have limited time for continuing education, research, or exploration of emerging technologies. Clinicians are motivated to learn more, but they need guidance through organization-led directives. Rural health care institutions should consider investing in mHealth analysis, tool development, and formal recommendations of sanctioned tools for clinicians to use with patients.

## Introduction

### Background and Significance

Out of an unprecedented adoption of mobile communication technologies and the progressive advancement of their application to personal and population health management, a new field of science, research, and health care has emerged—the study of mobile health (mHealth). The World Health Organization defines mHealth as “medical and public health practice supported by mobile devices, such as mobile phones, patient monitoring devices, personal digital assistants (PDAs), and other wireless devices” [1]. Evolving in recent years, the Knowledge for Health project describes the discipline by stating, “mobile health, or mHealth, is broadly the use of mobile and wireless technologies to support the delivery and utilization of health care services.” mHealth is a young field, and a limited evidence base exists for demonstrating its efficacy, effectiveness, and comparative effectiveness, especially its cost-effectiveness [2]. Both these definitions imply an organizationally driven or practice-driven approach to health care. In addition, the definitions suggest that the process is merely enhanced by the use of these new technologies.

Applications (apps) are software programs. Mobile apps are software developed specifically for a mobile device, such as a mobile phone or tablet. Native apps are software programs developed to be installed directly onto a device, typically downloaded through an app store. In this paper, mHealth apps include software accessible through websites and native mobile apps.

mHealth also includes wearable activity monitors (WAMs). Hundreds of WAMs are on the market and being used by consumers across a wide range of industries and occupations [3]. WAMs include a multitude of devices that are worn in and on various body parts or clothing, such as the wrist or pocket. Some companies offering these devices include Apple, Fitbit, Under Armour, Garmin, Jawbone, Pebble Time, LG, and Misfit [3].

The treatment of many chronic conditions takes place primarily outside the purview and physical environment of a doctor’s office. Although clinicians often require patients to recall detailed information about their symptoms and condition, the appointments may be spread apart, and recollection of specific situations can be challenging for patients [4]. Innovative mHealth apps provide the opportunity to enhance and improve the data collection and reporting processes, the patient-clinician interaction, and health outcomes [5-9]. Furthermore, the technology may make self-reporting easier for the patient and possibly more accurate and complete as well [10,11].

Some clinicians and organizations may argue that they should not base clinical decisions on patient-reported data. This study sought to uncover barriers to the use of patient-reported data and also motivators for clinicians who are already practicing in this manner. The opportunity exists for patient-driven health management and is readily available through a number of consumer software. The number of mHealth apps in publicly available app stores has grown exponentially in recent years to more than 325,000, with Android as the leading platform in 2017 [12,13].

iTunes and Google Play stores are the 2 largest in terms of apps available [14], as well as in number of available mHealth apps. iTunes is a publicly available app store where Apple device users can access software apps and directly install them onto their personal devices. Some apps are free, whereas others charge a fee for the installation, and some have a monthly or annual subscription [15]. Google Play is very similar, although the apps available in that store are for Android users. Although the public has access to an increasingly large number of apps, there appears to be limited traction among rural health care organizations to actively prescribe or engage patients with mHealth technologies, much less adopt and integrate patient-reported data into an existing electronic medical record (EMR). Therefore, despite the public’s easy access to hundreds of thousands of mHealth apps, they are not widely implemented by rural health care providers.

### A Framework for Readiness

In a rural community of Bangladesh, Khatun et al [16] surveyed 4915 randomly selected household members aged 18 years and older. The research team found that only 5% of participants had internet connectivity, only 50% were aware of SMS apps, and only 37% generally read them. Literacy was the primary barrier. In addition, 21% needed to charge their phones at someone else’s home, as there was no electricity in their own homes. Despite these barriers, the majority (73%) showed an interest in using mHealth technology in the future.

**Figure 1:**
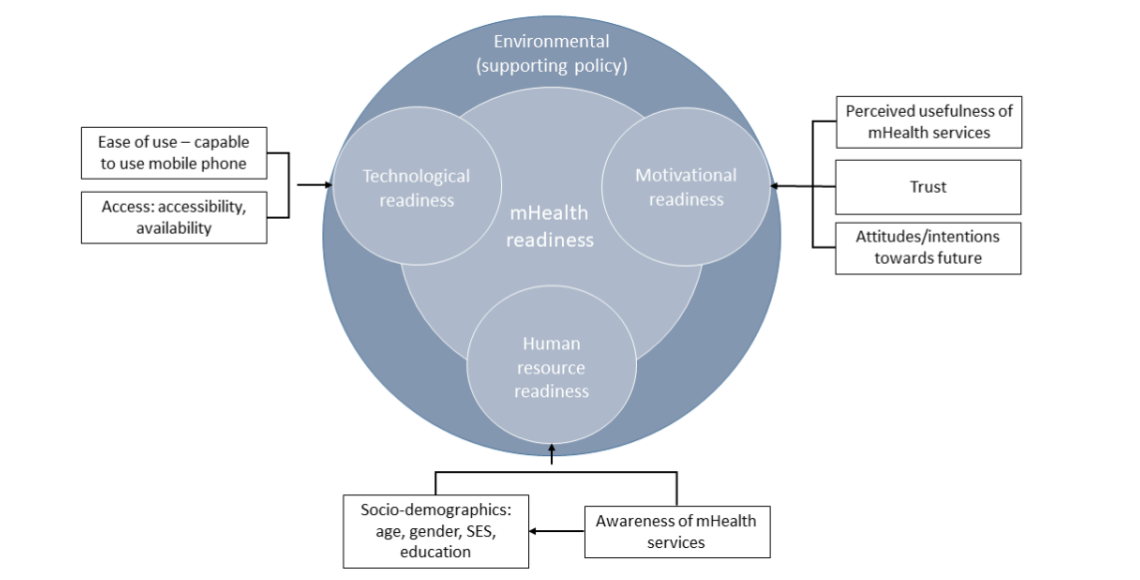
Conceptual model for mHealth readiness, developed by Khatun et al [16]. mHealth: mobile health; SES: socioeconomic status.

The team developed a framework for assessing community readiness for mHealth. The developed framework, further described herein and seen in Figure 1, has led to some interesting findings that have helped guide other studies, including this paper. The framework identified 3 high-level areas of readiness that are described here in greater detail. Furthermore, a model assessing clinician readiness was developed.

Previous literature is encouraging, but it should be noted that many of those studies were single interventions, often focused on isolated communities. There is currently a gap in the knowledge and literature around clinicians’ unofficial use of these types of technologies with rural patients. It may be that clinicians doing so are acting in silos with limited or no organizational direction, oversight, or approval processes. The purpose of this study was to create a model for assessing the necessary conditions for rural health care’s mHealth readiness through a qualitative assessment of clinician-perceived barriers.

## Methods

### Overview

mHealth technologies, such as apps, are widely available, along with the personal devices needed to run them. Clinicians and researchers have had successes with mHealth in urban and rural settings at various locations worldwide. However, limited organizational adoption has emerged in rural Wisconsin.

As a result, there are rarely formal policies and procedures to evaluate when assessing the use and impact of mHealth technologies. Therefore, this study team conducted semistructured, open-ended interviews with clinicians, as they were seen as the primary decision makers as to whether mHealth was used in patient care. Clinicians were recruited based on availability and not whether they were actively using mHealth technology. The open-ended nature of the interview allowed clinicians to explore their thoughts about using mHealth without assuming any prior beliefs or commitments on the part of the researcher [17]. These open-ended discussions provided qualitative data that were later used to refine a technology readiness model.

The 2-part methodology included consultations and interviews as the primary data collection and refinement of an mHealth readiness model using this study’s findings. Clinicians who see patients are particularly busy and may be unlikely to complete an electronic or paper-based survey without an incentive [18-20].

### Theory

The Diffusion of Innovation theory is one of the oldest social science theories and was developed by E.M. Rogers in 1962 [21]. The theory originated in communications, and the concept guided explanations of how ideas may gain in popularity and momentum to spread through a population or social system. This theory informed and guided the project and will be further assessed in its relation to the newly refined conceptual model for mHealth readiness, particularly as a framework for clinician and organizational adoption of mHealth technologies.

### Recruitment

Interviews and consultations were conducted at Marshfield Clinic Health System (MCHS), Wisconsin. Interviews were one-on-one interactions. Clinicians were recruited via phone and email, and interviews were scheduled at their convenience.

### Data Collection

Data collection was carried out between February and April 2016. Sessions were recorded using a battery-powered Olympus DM-620 audio recorder. Handwritten notes were also taken.

Consultation interviews were conducted as a way to refine the interview schedule. A total of 3 MD clinicians were interviewed as consultations for the project. Each represented a different clinical specialty and background.

The principal method of investigation was open-ended, semistructured interviews. The interviews were loosely bound around questions that asked informants to reflect on their participation in mHealth. Open-ended, semistructured interviews are largely conversation driven, and clinician time was a constraint. Thus, it was difficult to predict exactly which questions may come up with individual subjects and at what point in the flow and under what context. The initial proposal called for 10 interviews or until data collection reached the point of theme saturation.

### Coding: Identifying Themes and Categories

Following the consultations and interviews, additional notes were taken, notes were reviewed, and the audio recording was reviewed for additional note-taking and thematic analysis. The primary method of analysis was audio coding [22]. On the basis of the literature review and the first author’s experience, codes were identified. These experiences also formed the basis of the study and the research questions. Deductive reasoning techniques, coding qualitative data with pre-existing codes already in mind, were used to construct theme and category linkages.

The following existing theme categories, identified by Khatun et al in their study of barriers to rural community readiness to adopt mHealth [16], were used as a starting point: (1) technological readiness, (2) motivational readiness, and (3) human resource readiness. These was a natural fit for the evaluation of community readiness, particularly a rural community. The researchers in a study by Khatun et al were also interested in the individuals within that community and had surveyed residents, using door-to-door, in-person data collection methods.

The abovementioned categories were modified for this study to (1) clinician readiness, (2) patient readiness, and (3) organizational readiness. As noted, these 3 categories were identified through the first author’s experience in the field and through the literature review, including a study by Khatun et al [16]. The themes themselves were identified as relationships between the codes and the categories, such as the relationship between the organization and the legal/liability barriers to mHealth adoption, which is described in further detail in the Results section.

The review of the audio recordings allowed for inductive reasoning [23]—the possibility that other themes and subthemes would emerge. This process was used to identify additional themes.

### Mobile Health Readiness Model Refinement

The description of the work by Khatun et al [16] was also used here as the basis for the readiness model refinement. The refinement of the model was assessed before the interviews, and several new categories were identified. The refined model is focused on clinician readiness with data collected from clinician interviews. In addition to modifying category titles and influencers, each category was further assessed for thematic linkages to codes. Those linkages or relationships encompass the core of this study, and the recommendations are built into a newly refined model.

### Institutional Review Board Approval

This study was approved as minimal risk after review by the institutional review board of the University of Wisconsin-Milwaukee. The protocol has been granted Exempt Status under Category 2 as governed by 45 CFR 46.101(b). This study was also reviewed by the Office of Research and Integrity of Marshfield Clinic Research Institute and approved as minimal risk and exempt from further review (45 CFR 46.101(b)(2)).

## Results

The patient base of this study was primarily Marshfield, Wisconsin, and the surrounding area. Generally, their patients lived within a 20-mile radius of the medical center, with some exceptions.

### Interviews

The interviews uncovered a strong personal interest and passion for the participants’ patients, and the conversations exposed uses of the technology that are already in their practice or that they are considering for future patient care (see Table 1). There were 12 different clinical specialties represented. Given the small sample and the other reported demographics, for the protection of the participants’ anonymity, clinical specialties have been omitted.

Of the 13 clinicians interviewed, 12 were physically located at the Marshfield center, Marshfield, Wisconsin, and 1 was located in Eau Claire, Wisconsin, but they saw patients remotely through telehealth and in person in Marshfield and Eau Claire. Additional 10 clinicians were contacted during recruitment, but they were unable to participate. There were 5 female and 8 male participants. At the time of the interviews, 100% of participants owned tablets, and 92% owned mobile phones, and the 1 clinician who did not own a mobile phone was planning on purchasing one within the next few weeks. In addition, 77% were parents, and 30% of those were grandparents.

**Table 1:** Descriptive demographics of interview participants.

Process	Age (years)	Gender	Mobile phone owner	Tablet owner	Patients seen per month (n)	Percent of patients viewed as rural (%)	Prescribing/encouraging mHealth app/technology	Examples used or plan to use
Consultation	61	Female	Yes	Yes	Question not asked	Question not asked	Yes	Fitbit and MyFitnessPal
Consultation	62	Male	Yes	Yes	14-22	100	No	—^a^
Consultation	37	Male	Yes	Yes	200	10-20	No; but hopes to	Fitness
Interview	58	Male	Yes	Yes	200	90	No	—
Interview	37	Male	Yes	Yes	14-22	Most	Yes	MyFitnessPal
Interview	47	Female	Yes	Yes	100	95-100	Yes	Fitbit
Interview	43	Male	Yes	Yes	40	100	Yes	MyFitnessPal
Interview	47	Female	Yes	Yes	200	75	Yes	Omitted
Interview	53	Female	Yes	Yes	40	75	No; but hopes to	Blood pressure
Interview	40	Male	Yes	Yes	10	60	No; but hopes to	Physical Rehab
Interview	40	Male	Yes	Yes	200	100	Yes	Apple iWatch and Fitbit
Interview	46	Female	Yes	Yes	48	100	Yes	Blood sugar, fitness, and others omitted
Interview	37	Male	Yes	Yes	200	90	No; but plans to	Fitness

^a^Not applicable.

### Demographics

Although all participants were clinicians, few had an understanding of the term *mHealth* or its scope, with only 23% responding that they were familiar with the term before the interview. Although once described and examples given, all participants knew of other cases and gave examples of mobile devices or wearables used for personal health-related activities.

### Barriers

The analyses and coding of the data showed that barriers to adoption were one of the most important factors affecting clinician readiness to adopt. At this stage of the model refinement, we have focused on the issues of clinicians’ perceived barriers, further described in the sections below.

Table 2 describes the top barriers identified during the interviews and consultations combined. This question specifically asked, “Now that we’ve talked for a while, I want to ask again—what do you think are the top three barriers to clinicians using mHealth technologies with rural patients?” Several clinicians listed more than 3 barriers: this table only shows the top 3 responses tallied (the remaining answers were omitted). *Clinician familiarity* and *clinician time* accounted for 38.4% of identified barriers (15/39) to using mHealth apps or technologies with rural patients. Of the next 10 barriers, 8 were organizationally based, including EMR/data with 12.8% (5/39), and Health Insurance Portability and Accountability Act/Protected Health Information (HIPAA/PHI) with 10.2% (4/39) of total responses. Patient connectivity was combined with technology adoption and accounted for 10.2% (4/39) of the total responses, which was higher than anticipated.

**Table 2:** Top three barriers to mobile health adoption, identified during interviews and consultations (n=13; 39 responses).

Barriers	Responses, n (%)
Clinician familiarity	9 (23.1)
Clinician time	6 (15.4)
Electronic medical record/data	5 (12.8)
Health Insurance Portability and Accountability Act/Protected Health Information	4 (10.1)
Patient connectivity/technology	4 (10.1)
Organizational direction	2 (5.1)
Patient acceptance	2 (5.1)
Patient affordability	2 (5.1)
Uniformity of use	2 (5.1)
Hindering patient-provider communication	1 (2.6)
Technology reliance and limited patient face time	1 (2.6)
Usability of the app/technology	1 (2.6)

### Clinician Adoption

As shown in Table 1, 53.8% of participants reported encouraging the use of apps or WAMs with patients. When discussing MyFitnessPal, one clinician said the following:

I’ve had a fair amount of success by cueing people into some things like My Fitness Pal. If I’ve assessed a patient and they have some readiness to change, I’ll do some motivational interviewing around that and get people to buy into it and committed to making a lifestyle change. I’ll specifically call out a tool to help them. I’ve had the patient pull it up in the app store and download it in the office and kind of get them started in terms of what you need to do. Both I and my spouse have used that tool in our practices and have had patients that have had tremendous results because of it.

According to the participant, the couple had heard a speaker giving a presentation on this mHealth tool at a continuing medical education event, and also noted the following:

...it wasn’t driving patients to alter drug therapy; it was a way to track, record, and provide feedback. So, it was just a great way to engage patients. My spouse and I started using it, and then started recommending it to patients.

Although the participants did note limitations and that it is not a one size fits all approach, they described notable successes and a positive outlook on the technology and its use in patient care:

Does it help everybody? No. But a much higher percentage of patients had a positive change as a result of using that, than I ever saw had a positive change by me speaking with them and giving them a pamphlet to take home. It gave them something actionable, something that they had to report into. And when they would come back in, they would actually pull it up on their smartphones. We could kind of walk through and I could see they were actually doing it and we were seeing the results, in terms of what their numbers looked like.

This participant compared the data collection with a patient’s home blood sugar monitoring:

I don’t go through all of those [data], I look at their average or I look at a range and say their blood sugar over the past two weeks ranged from fasting morning sugars of 100 up to 150 and I record things more globally that way.

Although noting that he/she does put some of the patient-reported data into the EMR, he/she did prefer to have some type of automatic feed.

#### Clinicians’ Perceived Barriers Category 1: Personal (Clinician)

This category was established as a division of themes relating back to the clinician’s personal barriers and not necessarily influenced by the organization or the patient. Of the 3 categories, this category yielded the smallest number of responses.

##### Personal/Clinician: Familiarity With Mobile Health Options

Although a majority of the participants reported engagement with mHealth on a personal level, a lack of familiarity with appropriate and effective clinical apps of mHealth apps and technologies was common. This also was the leader in the *top three barriers* question, with 23% of all possible answers being *familiarity*. In addition, 66.6% (2/3) of consultations also reported this barrier, including one who stated that “taking the time to learn about what apps might be useful” is a challenge for them.

Unless they were a personal adopter, mHealth technologies were not something these clinicians were actively looking for, including trying to find new tools that might help with their clinical practice. Of those that were not currently using mHealth or WAM technologies with patients (46.1%), familiarity was one of the biggest barriers. One clinician was quoted saying:

How do you learn about it? To me that’s a barrier. It’s a time issue. I’m not going to spend my free time looking for it. And if the clinic doesn’t offer it to me I’m probably not going to learn a lot about it. If someone put on a continuing education course on mobile apps that you can use with your patients, I would probably go, but I have never seen one. There’s never been one offered here. I’m not sitting around at my desk a lot in my medical practice doing nothing, where I would have time to look these things up.

#### Clinicians’ Perceived Barriers Category 2: Patient

One clinician who spoke at length about patient barriers was also an adopter of mHealth and frequently promoted the use of several technologies with her patients. The majority of the patient barriers she identified during the interview were based on experience, not speculation. Those barriers are described in this section, although these barriers were not highlighted across all interviews or specialties. The data are not exclusively described in a way in which the patient barriers are assumptions based on speculation of the clinician or based on actual attempts to implement mHealth technologies. The results described here are a mix of both.

##### Patient: Affordability

Patient affordability emerged early on but was limited, and it was mentioned only once in the *top three barriers* of clinicians’ question, although it was discussed during 2 other interviews as a potential barrier. Affordability does not appear to hold a strong argument for barriers to adoption. An assumption is that this was likely a more significant barrier 5 to 10 years ago as the mobile phone adoption rates were just starting to climb.

##### Patient: Willingness and Adherence

Coded as a patient acceptance response to the *top three barriers* question, the willingness and adherence of patients were discussed with 2 participants. One of those participants did use mHealth technologies with his/her patients, but he/she also saw a unique patient population not representative of the whole patient population. Although, as noted above, the specialty is omitted to protect the identity of the participant, in this case, further research with regard to barriers related specifically to the participants’ specialty is warranted.

##### Patient: Access and Connectivity

Access and connectivity was a barrier identified on 10% of the responses to the *top three barriers* question. It also emerged throughout the interviews. Although conversations touched on this topic at some point, it was not always in a way that access and connectivity were viewed as barriers. For example, one participant talked about access and connectivity as a barrier when they had first started to promote MyFitnessPal with patients. This was, in part, because many of the patients still had flip phones at the time and were encouraged to use their home computer to keep track of activity. Use of home computers had limited success. However, since the increased adoption of mobile phones among that participant’s patient population, the participant has seen this particular theme of access and connectivity fade as a barrier. Others also noted that access and connectivity is no longer a hurdle because cell coverage is so well spread across Marshfield Clinic’s service area.

#### Clinicians’ Perceived Barriers Category 3: Organizational

##### Organizational: Limited Time and Existing Knowledge

All 3 consultations noted time limitation as a barrier to understanding, locating, evaluating, or implementing mHealth technologies with patients. Time also accounted for 15.3% of the responses to the *top three barriers* question. Clinicians, particularly physicians, have tight schedules with very limited time for continuing education, research, or exploration into new technologies. One participant was concerned about the potential *time sink* that an app, which facilitates patient-to-physician interaction, might impose. When asked what he thought other clinicians’ barriers might be, he said:

Once I’ve initiated the use of the app, will I be overwhelmed by the amount of information that’s provided to me? More than I can handle, and at times disappoint the expectations of my patients because I can’t? You know, they’re emailing me or texting me, or doing whatever it is that this app is going to do, and I don’t have time to respond to all may patients who are using the app. That’s another potential barrier I think.

##### Organizational: Uniformity

A lack of uniformity across clinicians and departments emerged from the interviews. Uniformity in this context is described as a common and guided practice among different clinicians to use similar mHealth tools. Clinicians who commented on uniformity noted that it should be considered in any type of organizational model moving forward. Several participants noted they did not have any type of departmental process in place but felt they would benefit from some type of uniformity or common footing. This concept links closely with the next section, which more specifically defines an organizational piece that is missing.

##### Organizational: Lack of Policy/Direction

The tone of the interviews highlighted a number of different concepts and ideas that were not necessarily identified in the participants’ *top three barriers*. Lack of policy/direction emerged from every interview and consultation as a point of discussion. Although when asked the question of *top three barriers*, only 23% mentioned this as a top barrier. If asked, none of the participants were aware of a policy or specific process, either departmental or organizational, in regard to the review or screening of a new mHealth technology that a clinician would like to put into practice with his/her patients. As it stands, those that are currently encouraging patient use of some type of mHealth technology have done so with no authoritative oversight other than their own or sometimes peer recommendation and review. Nevertheless, of all the technologies mentioned, the majority were health and fitness related (eg, MyFitnessPal and Fitbit), with others that were more specialty focused (data omitted to protect participant anonymity).

Clinicians also discussed additional challenges of understanding organizational policy and procedures, especially regarding instances where precedence has not already been established. One conversation that followed a question regarding the process for integrating a new mobile technology led to one clinician stating that:

I came here and I now understand Dilbert cartoons, because you can’t just do it. In fact, in my first year I innocently tried to do a few things and got my hand slapped because I was going outside of channels and I didn’t even know channels existed yet and that wasn’t mHealth sorts of things, but other things.

The participant did note that this was in regard to another type of issue, not mHealth, but still held weighted and influenced the subjects’ perception of approval processes. Another participant noted that the act of “trying to get all the different people on board, and even knowing who to ask sometimes, is really difficult.”

In the absence of a formal written policy, the policy section can be further broken down into the following sections: (1) information systems security and related policies, (2) legal concerns with liabilities, and (3) app quality and patient safety. Surprisingly, none of these 3 sections were discussed by participants with any significance. Policy was discussed from an organizational direction perspective. Security was discussed, but from a patient data and patient privacy perspective, and legal concerns and liabilities were discussed and are further noted below. There were just 2 mentions of app/WAM quality, which are mentioned later in the paper, and there was no discussion about patient safety.

##### Organizational: Health Insurance Portability and Accountability Act/Protected Health Information

Responses that were combined into this code included *HIPAA*, *PHI*, *privacy, security*, *confidentiality*, and *patient privacy*. Clinicians were outspoken about this as a barrier to how they practice or how they would like to practice medicine, with 10.2% of responses in the *Top 3 barriers* question. One clinician noted that “HIPAA turns out to be a barrier, because everyone is so afraid of messing it up,” and another clinician referred to the general concept as *“*HIPAA-phobia” and often a prohibitive element within the health care industry. Another participant referred to HIPAA regulations as “very confusing and difficult to interpret.”

##### Organizational: Data and the Electronic Medical Record

Almost all the interviews and consultations voiced their desire to integrate patient-reported data into the EMR. Clinicians also noted that the current system would not be able to handle it in an efficient way. None of the participants commented in detail on the constraints and technological challenges of mapping patient-reported data to clinical systems or how best to store that data for later retrieval and how it would be used with clinical decision support. In summary, clinicians are already using that data and would like to have them somehow integrated into the EMR.

When talking with one of the participants about the current Cattails MD and My Marshfield Clinic System that is used at MCHS, the clinician was quoted saying:

We have to learn how to leverage tools like that, in concert with some of these apps and other things, to say how can we exchange information out of your Fitbit that helps connect your care team with how you’re doing? Are you getting your steps every day? Are you on track with your caloric intake and all of that stuff? So, I think there’s some opportunity to marry those through a tool like the Portal.

Another participant who was interested in using that data noted he/she often would make a note of using the MyFitnessPal with the patient, but there is no structured or uniform method of capturing such data. When asked about the process of recording it, the clinician noted that he/she did not transfer detailed data such as weights, steps, and activities, “mostly because it’s just an onerous task to transcribe those over.”

##### Organizational: Legal and Liability

Legal refers to the Legal Department within the organization. As just a code, legal/liability holds little meaning and could be easily linked to a clinician barrier, as personal liability or malpractice; that particular linkage was only identified as a barrier during the interviews once. One clinician noted that “some are afraid that by recommending something, if anything ever went wrong, that they would somehow be personally liable.” Meanwhile, the linkage between the *organization* and legal/liability was more frequent.

All 3 consultations noted specific organizational constraints in regard to using mHealth apps and technologies with patients. Of the 3 consultations, 2 cited *legal* as one of the barriers in trying to get an organizational decision. One of the consultations suggested departmental consensus and recommendations as a way to move new techniques through legal and into practice.

### Thematic Map

**Figure 2:**
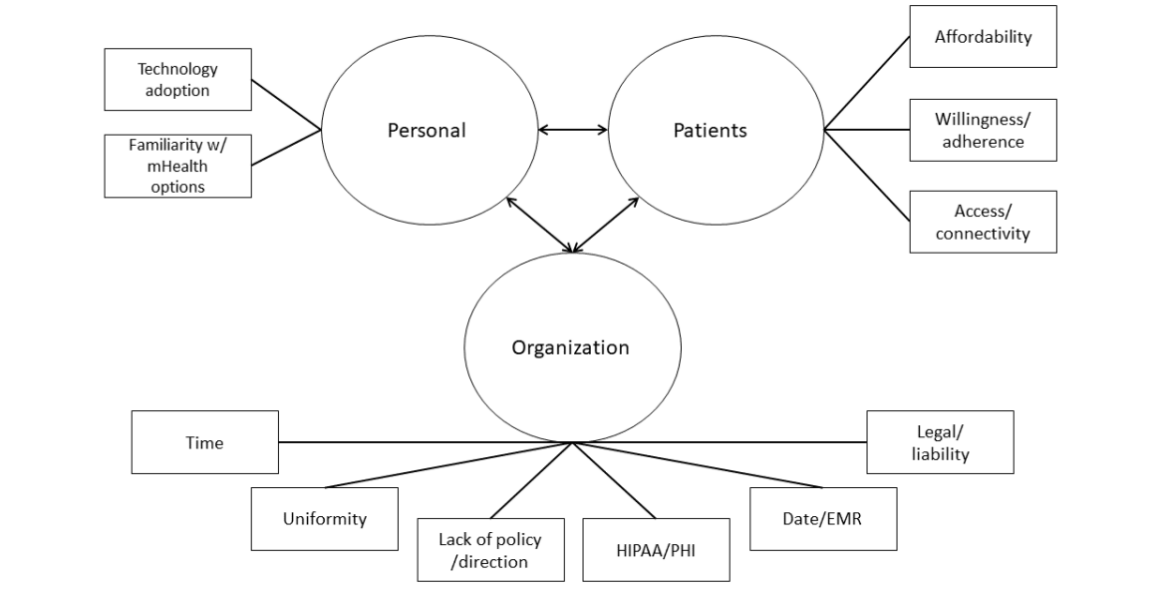
Thematic map of clinician-identified barriers. EMR: electronic medical record; HIPAA/PHI: Health Insurance Portability and Accountability Act/Protected Health Information; mHealth: mobile health.

Figure 2 displays the coded responses of the top barriers identified throughout the interview process as well as the thematic placement in identified categories of personal, patients, and organizational.

### Mobile Health Readiness Model Refinement

The original model of mHealth readiness was based on a study by Khatun et al [24]. As the refinement process began, 3 pillars and primary dimensions of readiness were formed: technological, motivational, and clinician. *Technological* is primarily the technical ability for the clinician and his/her patients to be able to connect and engage with an mHealth app. Both would need to have a device (eg, mobile phone or tablet), connectivity (home for patients, office/examination room for clinicians) through mobile broadband, and/or connectivity through broadband (eg, Wi-Fi). This study did not seek to assess the connectivity and overall readiness of the patients, just the perceived patient readiness in the opinion of clinicians. *Motivational* refers to the willingness of clinicians to engage with patients in mHealth. Clinician and organizational readiness are the key areas of inquiry for this project; thus, the refinement began.

**Figure 3:**
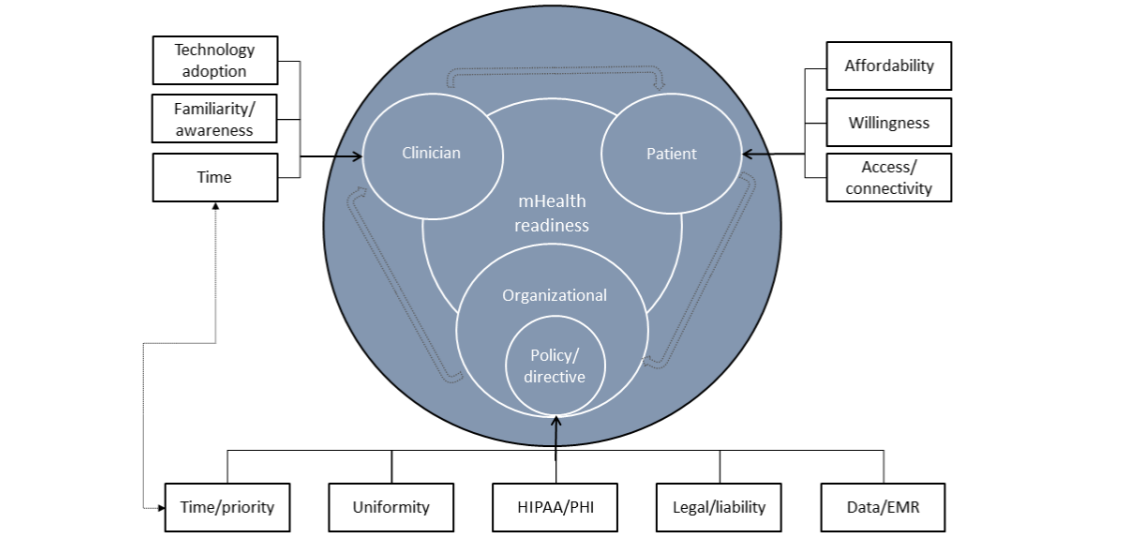
Conceptual model for assessing necessary conditions for rural health care’s mHealth readiness, with an emphasis on clinician-perceived barriers. EMR: electronic medical record; HIPAA/PHI: Health Insurance Portability and Accountability Act/Protected Health Information; mHealth: mobile health.

As the interviews unveiled thematic linkages between codes and categories, the framework refinements began to take shape as the model displayed in Figure 3. The technological pillar was eliminated early, as the data showed this is a very small barrier, if at all. The motivational barrier was also eliminated from the framework once enough data showed that clinicians who participated in this study were adopters themselves and promoters of mHealth technologies with patients.

This is a framework that has not yet been tested. The model should be used with the assumption that patients and clinicians are already motivated and that technology is not a barrier to adoption. As both those pillars were eliminated from the original model, they are not to be included in assessments using the new model.

The coded barriers form a basis of decision making and readiness for each of the 3 categorical pillars, now labeled as *clinician*, *patient*, and *organizational*. These 3 primary dimensions have been defined as pinnacle and foundational elements of assessing overall readiness to adopt mHealth in patient care. The double-wide arrows represent an informational flow from organization to clinician to patient and back to the organization in the form of feedback and patient experience. This framework was developed for MCHS specifically and for other health care organizations generally.

There is an arrow connecting *time* from the clinician and the organizational categories. This represents a unique barrier that can be placed in either or both of the categories. For example, the clinician’s time was a clear barrier to adoption, but it is not known if that is an organizational barrier related to not enough time allowed in the schedules for research/education. The other complexity is that time and priority are potential barriers for the organization itself. Owing to the current cultural climate and the competitive expansion into new markets, including the acquisition of a hospital and its thousands of staff members, the organizational priority and available administrative time to dedicate to mHealth may be limited.

Although created based on international work, the model may not be as easily adaptable to international health care organizations. It could possibly serve as a framework, but it would have to be thoroughly tested, as there is so much variation in external variables and the potential for different barriers in each organization, region, or country.

## Discussion

### Principal Findings

This project sought to understand the barriers to adoption, with a strong assumption that some innovative clinicians were already using mHealth tools with rural patients. This assumption was confirmed during the interview process, and in fact, an even higher rate of adoption was found than anticipated. The small sample size of our study is not statistically significant, and it is likely not representative of the entire MCHS clinician body. Nevertheless, our results strongly suggest that health care organizations should consider investing in mHealth analysis, tool development, and the promotion/recommendation of sanctioned tools for clinicians to use with their patients.

Many studies have investigated effectiveness and progress of mHealth adoption, particularly in developing and low-income countries [25-29]. Yet, much work remains to be done in the United States, particularly among rural health care organizations, which is a rapidly changing landscape as mergers, acquisitions, and hospital closures have shaken up the industry in recent decades [30,31].

Organizational mHealth readiness depends on a number of factors, not all of them addressed in this study. However, the readiness of clinicians was identified, as well as barriers preventing them from furthering their mHealth practices. A follow-on study could help to ascertain the perceived relative importance of the barriers identified. For example, it will be useful to discover whether *time* and *familiarity* fall under personal/clinician or under organizational category. It could be argued that the organization is responsible for the education, training, and communication about new technologies for its physicians and staff. It could as well be argued that the clinician is responsible for keeping abreast of new opportunities, including mHealth, for improving patient care.

One of the initial consultation interviews was with a participant who was very interested in the project and what our team might learn about mHealth adoption. Near the end of the interview, the author described more about some of what researchers and clinicians have been using, as described in the literature. The participant was very interested, saying, “you’ve been holding that back this whole time?” The interview schedule, as described earlier, was semistructured. Later in the interview process, as in this case, there was often additional discussion about what falls within the realm of mHealth and what some clinicians and organizations have had success with, as available in the scientific literature. The clinician also said, “these are things that we should start talking about as a group, immediately. These are inspiring.”

The same clinician continued to describe some of the possibilities where mHealth would be useful. The clinician then added, “I think about maybe kids that are in that tween to teenager years that are having challenges to get more active and curb their weight, lose weight, do something like that. I’m inspired; I think I should start something like this. That would be great.” The author had not planned on discussing mHealth as a way to influence the participant in any way. The tone of any information was neutral, as much as possible, so as to not persuade or motivate the participant to consider mHealth technologies. Limitations, challenges, and barriers were discussed, as well as some of the interventions in the literature. This particular physician’s reaction is another example that suggests health care organizations should consider investing in mHealth adoption.

Other interviews yielded similar results and further showed that the organization is not yet prepared for innovative clinicians. mHealth as a tool for improving patient outcomes is already being embraced by some, and others may be very interested in its potential. One clinician’s perceived value was described as the time between visits, which is otherwise unmonitored and loosely reported with unknown accuracy during the next appointment. That clinician commented:

Where I see the real value, is in between visits. So, I may only see a patient with diabetes every 6 months or see a patient with high cholesterol once a year. So, you might cue them in to My Fitness Pal and talk about lifestyle modifications. But do I bring them back in two months specifically to say, are you using your app? No. If there’s no clinical reason to bring them back and it’s just preventative care, you might not see that person for a year. But if that was then connected and you have the ability to make that link you could actually have some tracking of patients’ in-between time and provide some positive reinforcements. So let’s say the patient starts using a tool like My Fitness Pal and they hit a ten pound weight loss. Well, that would be great if that could somehow trigger onto my worklist so that our practice could do an outbound communication to that person as a “thumbs up” you hit the ten pound weight loss! That’s awesome! Let us know how we can help. Let people know that you’re involved in their care and you’re recognizing and applauding them for their efforts and helping to fuel the fire.

Although there are some risks that would need to be managed and mitigated, if organizations were prepared to manage mHealth, it is very likely that clinicians could improve the quality of care for their patients. Our preliminary results suggest that many physicians may see such benefits of mHealth technologies. However, many organizations, including MCHS, are not yet prepared to prescribe or prohibit the use of mHealth technologies. Having said that, there are some recommendations that come from this study, particularly the formation and promotion of a Resource menu. Those details are further described in the section Unexpected Outcomes.

### Unexpected Outcomes

The project yielded a number of unexpected outcomes from the interviews. These outcomes suggest potential future projects as well as immediately practical actions to take based on recommendations from the participants in this study. Outcomes are also discussed in the section below.

#### Mobile Health Resource Menu

Participants showed a desire for guidance and instruction, especially with regard to recommendations on what to use with patients, what others are having success with, and what has positively affected patient outcomes. Several participants noted that a seminar, webinar, or presentation on mHealth apps would be helpful, and they would attend. Participants were interested in a *menu* that offers the organization’s recommended mobile apps or technologies. One of the participant’s responses included:

I would love to have some sort of databank of vetted apps that we could recommend for our patients, without each and every one of us just playing around and trying to figure it out for ourselves. To say that, if you’re going to motivate patients for weight loss, here are some good apps that have been looked at by the organization, are as free of bias as you can be, and safe. I know there has been some discussion, and once in a while I hear bits and pieces, but I don’t think there’s anything formal in place.

After several subjects requested this type of document during interviews, it was added to the schedule as a question for others. Of those who were asked if they would be interested in seeing such a list, all the participants said they would. One was quoted saying:

I think having some of that stuff sanctioned and clarified for our providers who are generally mystified by all of this, because most of them only know what they’ve taken initiative to learn on their own. And as an organization, I think we can do a lot better job of steerage and helping people to go through those thousands of different options and say here’s what we consider to be the cream of the crop. And give providers, and maybe even patients, a library of things that they could go to that might be helpful for them.

Although app and technology quality never arose as a solidified theme, some clinicians hinted to it in ways such as this participant’s comment:

I don’t think we want to promote an app that is a 30-day grapefruit diet. So who’s going to vet all that stuff when the world of apps is just exploding. I think it’s a real issue for us.

A quality review of mHealth technologies certainly needs to be a part of any type of formal vetting and recommendation process. 

#### Accuracy and Honesty in the Data

The honesty of participants is often critiqued in qualitative research, questioning the integrity of participant responses, and thus the data. This study was able to eliminate some of that critique through the context that bookended said responses. Participants were honest about experiences that they would have been behooved to lie about. For example, the use of unsanctioned apps and technologies in patient care was described at length by clinicians who knowingly were practicing outside the purview of higher authority, guidance, or direction. Therefore, it seems as though all participants were providing candid responses.

### Limitations

Clinicians noted they were using various mHealth technologies with patients, and they also expressed excitement of those interventions’ results. Several examples were intentionally omitted from this paper because of the possibility of identifying participants through their clinical specialty and the specificity of the mHealth apps being used with patients. Multiple institutions and a broader geographic reach for participant recruitment would be ideal. Not only would data be more representative of the population, we would also more descriptively report the findings. Specifically, we could have described participants’ clinical specialties and the unique technologies and apps that are being used therein. Those identifiers were stricken from this paper.

### Sample Size and Time of Clinicians

Recruitment was a challenge, as is often the case in health care research [32]. Clinicians, particularly physicians, have a demanding schedule and are often tasked with seeing as many patients as possible throughout their workday. Even the nonphysician staff who were contacted (eg, physical therapists, clinical psychologists, and nurse practitioners) were either nonresponsive or not able to accommodate a 45-min to 1-hour interview. Those who did participate were interested in shortening it during the scheduling process. Several interviews were interrupted for a patient care issue that needed to be addressed, but they were reconvened minutes later. The only participants who were not interrupted had dedicated research time as part of their contract, were currently traveling, or were meeting at the end of their workday.

### Conclusions

Mobile technologies are not often restricted by geographic boundaries or distance, traffic conditions, or weather. On the basis of these factors alone, rural populations may have more to gain through the use of these technologies. Beyond rural, the application of these technologies may also be critical to other populations, such as parents with two jobs who have a difficult time taking vacation for appointments.

Research needs to further assess organizational/administrative perceptions, which may include interview with legal teams and decision-making administrative leaders of health care organizations. What has been found through this research and a review of the literature is that clinicians are interested in or already using mHealth technologies with patients. In addition, patients are interested in or already using mHealth technologies, and some research has shown positive outcomes from mHealth interventions. Meanwhile, health care organizations have not yet actively embraced or supported its use. Organizational leadership should review these studies and the results so that they can make more informed decisions to proceed with formalized and sanctioned adoption of secure and safe evidence-based mHealth tools. Removing the identified barriers is necessary for adoption, but not sufficient for successful implementation or readiness.

## Abbreviations

EMR: electronic medical record
HIPAA: Health Insurance Portability and Accountability Act
MCHS: Marshfield Clinic Health System
mHealth: mobile health
PHI: Protected Health Information
WAM: wearable activity monitor
